# Investigation of Grain Boundary Effects in Sm_0.2_Ce_0.8_O_2−x_ Thin Film Memristors

**DOI:** 10.3390/ma17133360

**Published:** 2024-07-08

**Authors:** Weikai Shi, Luyao Wang, Nan Yang

**Affiliations:** School of Physical Science and Technology, ShanghaiTech University, Shanghai 201210, China; shiwk@shanghaitech.edu.cn (W.S.); wangly2@shanghaitech.edu.cn (L.W.)

**Keywords:** memristor, Sm_0.2_Ce_0.8_O_2−x_, pulse laser deposition, grain boundaries, oxygen vacancy

## Abstract

Cerium-based materials (CeO_2−x_) are of significant interest in the development of vacancy-modulated resistive switching (RS) memory devices. However, the influence of grain boundaries on the performance of memristors is very limited. To fill this gap, this study explores the influence of grain boundaries in cerium-based thin film resistive random-access memory (RRAM) devices. Sm_0.2_Ce_0.8_O_2−x_ (SDC20) thin films were deposited on (100)-oriented Nb-doped SrTiO_3_ (NSTO) and (110)-oriented NSTO substrates using pulsed laser deposition (PLD). Devices constructed with a Pt/SDC20/NSTO structure exhibited reversible and stable bipolar resistive switching (RS) behavior. The differences in conduction mechanisms between single-crystal and polycrystalline devices were confirmed, with single-crystal devices displaying a larger resistance window and higher stability. Combining the results of XPS and I–V curve fitting, it was confirmed that defects near the grain boundaries in the SDC-based memristors capture electrons, thereby affecting the overall performance of the RRAM devices.

## 1. Introduction

With the emergence of the Internet of Things (IoT) and cloud computing, there has been an exponential growth in data volume [1]. However, the classical computing architecture based on the von Neumann model exhibits fundamental limitations due to the so-called von Neumann bottleneck, which prevents simultaneous processing of data [2,3,4]. Memristors, as a type of non-volatile memory device based on RS, are regarded as an effective solution to overcome this issue due to their advantages, such as long data retention time, simple structure, high density integration, low power consumption, fast operation speed, strong scalability, straightforward components, and ease of integration with standard metal−oxide−semiconductor (MOS) technology [5]. Oxides are widely used in various fields due to their advantages, such as room temperature stability, multifunctionality, renewability, and processability [6]. In recent years, numerous oxide materials have been discovered to exhibit RS behavior, including SiO_2_ [7], TiO_2_ [8], Ta_2_O_5_ [9], HfO_2_ [10], BiFeO_3_ [11], and SrTiO_3_ [12], among others. In general, the switching of memristors is caused by the formation and disruption of conductive filaments, which can typically be classified into the following two cases: metal cation pathways and oxygen vacancy anion pathways [1].

CeO_2−x_, a classic fluorite-structured oxide, is widely investigated due to its considerable oxygen ion conductivity, structural stability, and other properties [13,14,15]. Introducing trivalent ions dopant can induce charge compensation defects in CeO_2_, such as oxygen vacancies and Ce^3+^, thereby enhancing the conductivity and oxygen storage properties of doped cerium (Do_x_Ce_1−x_O_2−y_) [16]. Although the formation of conductive filaments in memristors based on the VCM (Valence Change Memory) mechanism is not yet fully understood, most studies indicate a close association with oxygen vacancies [17,18,19]. Consequently, Do_x_Ce_1−x_O_2−y_ (e.g., SDC, GDC) is a potential material for the RS layer in RRAM. Recently, extensive studies have been conducted on thin film CeO_2−x_ as the RS layer. Ismail et al. studied the RS characteristics of Ti/CeO_2−x_/Pt devices with varying RS layer thicknesses and found that the electroforming voltage and set voltage decrease with decreasing film thickness. Rupp et al. discovered that Pt/Gd_x_Ce_1−x_O_2−y_/Pt devices with a doping concentration of 20 mol% exhibit optimal RS performance, while too low or too high doping concentrations have negative effects [20,21]. In CeO_2−x_, in addition to oxygen vacancies, the impact of planar defects, such as grain boundaries, are also crucial, as they significantly influence catalytic performance, electrical conductivity, and the oxygen storage and release capabilities [22,23]. However, the grain boundary effects in SDC thin film memristors have not been extensively investigated. Therefore, to fill this gap, we fabricated single-crystalline and polycrystalline SDC thin film memristors and analyzed the effects of grain boundaries in these devices.

Pulsed laser deposition (PLD) provides an effective method for the precise control of the microstructure of target materials, taking device miniaturization into consideration. Recent studies have utilized PLD for the microstructural manipulation of target materials. For instance, Dou et al. utilized temperature engineering to achieve three distinct grain morphologies in CeO_2_ thin films and demonstrated that devices with a “columnar scaffold” morphology exhibited the best overall resistive switching performance [24]. Additionally, Wang et al. used PLD to grow CeO_2_(111) unit cells on STO(110) substrates, which have a high lattice mismatch, resulting in CeO_2_ thin films with a significant number of grain boundaries [25]. Therefore, PLD is an effective method for defect engineering in cerium dioxide thin films, capable of introducing planar defects within the films. This provides an ideal platform for investigating the impact of grain boundaries on memristors.

This paper aims to explore the impact of grain boundaries on SDC thin film memristors. Using PLD, we grew both single-crystal and polycrystalline SDC films on (100)-oriented NSTO and (110)-oriented NSTO substrates, respectively. We investigated the bipolar resistive switching characteristics of devices with a Pt/SDC/NSTO structure, finding that single-crystal SDC film devices exhibited superior switching characteristics. Additionally, combining XPS and I–V curve fitting confirmed the differences in electron transport properties between single-crystalline and polycrystalline devices. Although atomic vacancies [26] enable CeO_2_ to perform resistive switching, grain boundaries have a negative impact. This evidence is important for defect engineering and provides new insights for the development of advanced doped cerium-based RRAM devices in the future.

## 2. Materials and Methods

### 2.1. Synthesis of SDC20 Powder

CeO_2_ and Sm_2_O_3_ powders were placed into a ball milling jar at the stoichiometric ratio, along with an appropriate amount of water as a dispersant, leaving approximately one-third of the space empty. Subsequently, an appropriate amount of ZrO_2_ spheres was added as the grinding media. Two similarly weighted ball milling jars were then placed into a ball miller with a rotation speed set at 30 Hz and milled for 12 h. Upon completion of milling, the resulting precursor powder was dried in an oven. The dried powder was then placed in a muffle furnace and calcined at 1000 °C in air for 5 h at a heating rate of 5 °C/min.

### 2.2. Thin Film Preparation of the Resistive Switching Devices

Single-crystal (100)- and (110)-oriented SrTiO_3_ substrates doped with 0.7 wt.% Nb, measuring 5 × 5 mm^2^, were subjected to ultrasonic cleaning in acetone and ethanol prior to loading into the UHV (ultra-high vacuum) chamber. Subsequently, SDC20 thin films were deposited on the substrates via pulsed laser deposition (NanoPLD, PVD Products, Wilmington, MA, USA) under a base pressure lower than 5 × 10^−7^ Pa and without any gas environment. SDC20 targets were prepared using conventional sintering methods. Plasma was generated between the substrate and target using a focused KrF excimer laser (wavelength: 248 nm) with a laser energy of 0.5 J/cm^2^ and a repetition rate of 10 Hz. Prior to SDC20 deposition, the substrate underwent in situ annealing at 650 °C to achieve a clean and flat surface. During the deposition process, the substrate was maintained at 650 °C. After deposition, the temperature was decreased to room temperature at a rate of 30 °C/min.

### 2.3. Resistive Switching Device Fabrication

Platinum (Pt) top electrodes (TE) were deposited at room temperature using radio frequency magnetron sputtering (Lesker ProlinePVD75, Kurt J. Lesker, Pittsburgh, PA, USA). The area of the TE was 100 × 100 μm^2^, and a mask was employed for patterning. Additionally, Pt was also used to contact the NSTO bottom electrode (BE).

### 2.4. Electrical Characterization of Resistive Switching Device

In this study, all electrical characterization measurements were conducted using an Agilent B1500 semiconductor parameter analyzer. Measurements were performed by contacting the electrodes using probes, with the top electrode biased positively and the bottom electrode grounded.

## 3. Results and Discussion

### 3.1. Powder Characterization and Analysis

The powder XRD analysis, as shown in Figure S1, reveals the formation of a cubic fluorite structure in the powder, with no apparent impurity phases. A comparison with the pure CeO_2_ structure indicates a slight increase in the 2θ diffraction peak of CeO_2_(220) from 47.46° to 47.67°, accompanied by a reduction in the c-axis lattice parameter. This reduction is attributed to the substitution of Sm^3+^ (0.958 Å) for Ce^4+^ (0.97 Å), leading to lattice contraction, consistent with the expected results. The doping of 20% Sm was further confirmed by Inductively Coupled Plasma Spectrometer (ICP) analysis of the powder composition, as shown in Table S1.

### 3.2. Characterization and Analysis of SDC20 Thin Films

**Figure 1:**
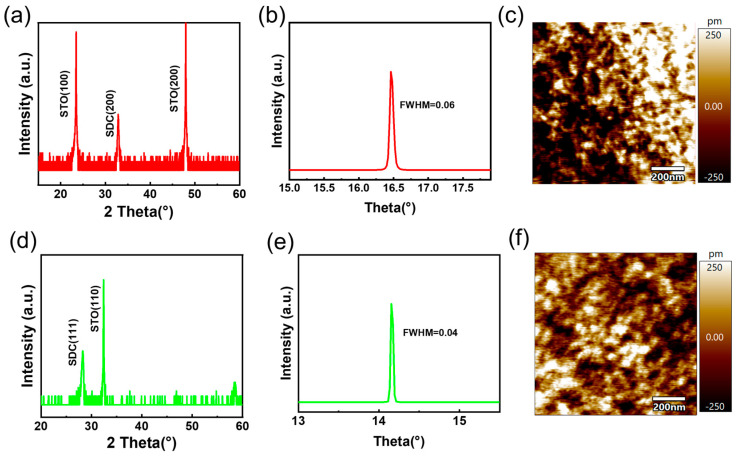
X-ray Diffraction (XRD) Spectrum and Atomic Force Microscopy (AFM) Images of Single-Crystal and Polycrystalline Devices: (**a**,**d**) high-resolution XRD spectrum of the SDC20 thin film. (**b**,**e**) Swinging curve at the (200) peak position of the SDC20 thin film. (**c**,**f**) Two-dimensional AFM image of the SDC20 thin film.

Figure 1a shows the X-ray diffraction (XRD) θ-2θ scan spectrum of the SDC20 film grown on (100)-oriented NSTO. According to the XRD spectra, only reflections corresponding to the (200) planes were observed on the SDC20 film, indicating that the SDC20 film is predominantly oriented along the c-axis. Figure 1d shows the X-ray diffraction (XRD) θ–2θ scan spectrum of the SDC20 film grown on (110)-oriented NSTO. Using Bragg’s formula, we determined the lattice parameters of single-crystalline and polycrystalline SDC thin films along the c-axis to be 5.44 Å and 5.46 Å, respectively. The greater distortion along the c-axis in polycrystalline SDC thin films indicates the presence of more defects within the bulk of the polycrystalline films. Figure 1b,e depict the rocking curves of the single-crystal and polycrystalline SDC20 films, respectively. The half-widths are in the range of 0.04°~0.06°, indicating that both possess high crystal quality. Figure 1c and Figure 1f, respectively show the two-dimensional AFM images of the single-crystal and polycrystalline SDC20 thin films. Both exhibit dense and smooth surfaces with uniform grain sizes and orderly arrangements. The AFM results show that the surface roughness of single-crystal and polycrystalline films is essentially the same, indicating that the contact between the Pt electrode and SDC is consistent in both single-crystal and polycrystalline devices. As shown in Figure S2, the surface morphology of single-crystal and polycrystalline SDC20 films was observed under a magnification of 50,000 times. Consistent with the AFM results presented in the previous section, both exhibited smooth and dense surfaces, indicating good film growth.

### 3.3. Electrical Characterization of the Devices

**Figure 2:**
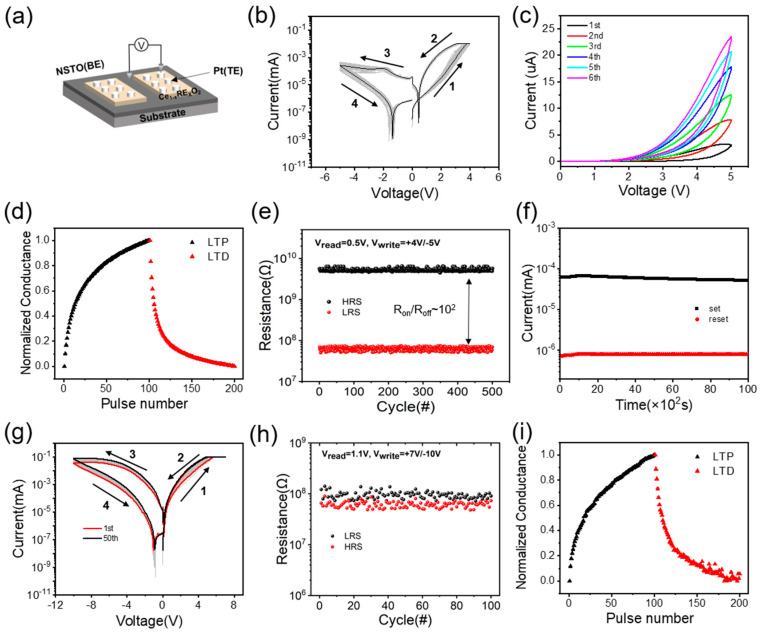
Electrical performance characterization of single-crystal (**a**–**f**) and polycrystalline (**g**–**i**) devices: (**a**) Schematic diagram of the device structure. (**b**) Typical I–V characteristic curves over 50 repeated switching cycles. (**c**) Multi-step set process of the device under a +5 V scanning voltage. (**d**) Synaptic testing of the device. (**e**) Endurance test over 500 consecutive reset-set cycles, read at 0.5 V. (**f**) Retention tests for HRS and LRS after reset and set operations, respectively, read at 0.5 V. (**g**) Typical I–V characteristic curves over 50 repeated switching cycles. (**h**) Endurance test over 100 consecutive reset-set cycles, read at 1.1 V. (**i**) Synaptic testing of the device.

Figure 2a illustrates the current−voltage (I–V) characteristics of Pt/SDC20/NSTO(100) devices measured in voltage sweep mode. Typical bipolar resistive switching (RS) behavior is observed over repeated switching cycles. Figure 2a provides a brief description of the device structure, wherein forward bias corresponds to the application of positive voltage to the top Pt electrode. Furthermore, our Pt/SDC20/NSTO(100) structure does not require a forming process. As shown in Figure 2b, the initial resistance of the Pt/SDC20/NSTO(100) device remains in a high-resistance state (HRS). When the voltage is swept from 0 V to +4 V and back to 0 V, hysteresis in the I–V curve is observed, indicating that the device has been set to a low-resistance state (LRS), i.e., the set process. By applying a negative bias, the device can return to HRS, as indicated in ranges 3 and 4 of Figure 2b, i.e., the reset process. Obtaining identical I–V hysteresis over 50 consecutive cycles demonstrates reversible and repeatable RS behavior. Figure 2c illustrates the multi-step set process of the device. By consecutively applying positive scanning voltages of 5 V for six cycles, the current gradually increases with each step of the set process, demonstrating analog memristive device characteristics. This observation is further corroborated by subsequent synaptic plasticity tests, as depicted in Figure 2d. Under a read voltage of 0.5 V, the conductivity changes were sequentially recorded during 50 identical positive pulses (5 V, 5 ms) and 50 identical negative pulses (−5 V, 5 ms). The conductivity of the device gradually increased with the application of positive pulses, reaching saturation thereafter. In contrast, negative pulses led to a decrease in conductivity until saturation. These nonlinear transmission pulse-time-dependent characteristics bear a similarity to the pulse-time-dependent plasticity observed in biological synapses. As depicted in Figure 2e, the resistance ratio (R_HRS_/R_LRS_) between HRS and LRS at +0.5 V (~180) indicates the applicability of our RRAM device. Throughout over 500 switching cycles, no significant degradation or drastic fluctuations in the bistable resistance states were observed. Additionally, following the switching of the device to either the High Resistance State (HRS) or the Low Resistance State (LRS), the time retention capabilities of the device in both HRS and LRS were assessed under a reading voltage of 0.5 V applied every 10 s (Figure 2f). The resistance values of both HRS and LRS remained stable, with no decline observed within 10^4^ s, thus confirming the non-volatile nature of the device. Similarly, we also conducted electrical performance tests on the polycrystalline Pt/SDC20/NSTO(110) devices. Figure 2g plots the current−voltage (I–V) characteristics of the Pt/SDC20/NSTO(110) device measured in voltage sweep mode, displaying bipolar I–V hysteresis loops within the range of −10 V to +7 V. As shown in Figure 2h, unlike the single-crystal device, after 100 cycles at a read voltage of 0.5 V, the R_on_/R_off_ ratio is only 3. Similarly, as shown in Figure 2i, the polycrystalline devices also exhibited analog memristive characteristics, demonstrating potentiation and depression under 100 consecutive positive pulses and 100 consecutive negative pulses, respectively.

### 3.4. I–V Curve Fitting Analysis

**Figure 3:**
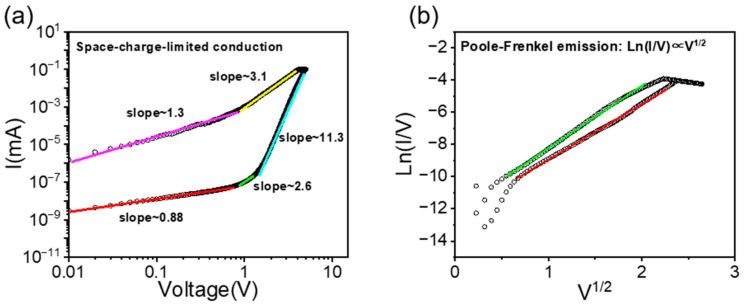
Fitting of I–V curves of the device in the positive voltage range with two different conduction models: (**a**) single-crystal devices: space-charge limited conduction and (**b**) polycrystalline devices: Poole−Frenkel emission.

To verify the conduction mechanisms of the devices, we fitted the forward I–V curves of two devices. As shown in Figure 3a, the forward portion of the single-crystal device was fitted according to the space-charge-limited current (SCLC) model. The SCLC model can be represented by the following equation [27]:

(1) $$J=\frac{9\mu \varepsilon_{r}\varepsilon_{0}}{8d^{3}}\left(\frac{\theta_{r}}{1+\theta_{r}}\right)V^{2}$$

where *J* is the current density, *ε* is the dielectric constant, *μ* is the carrier mobility, and *θ* is a defect-related constant. In the low voltage region, the slope is approximately 0.88, indicating a sub-linear relationship between current and voltage. This behavior may be related to ohmic conduction or defect state capture currents within the material. In the intermediate voltage region, the current exhibits a nonlinear relationship with increasing voltage, typically associated with the trap-filled limit (TFL) stage. As the voltage increases to a certain level, the trap states begin to fill up, leading to a rapid increase in current. In the higher voltage region, the current transitions to the SCLC region. In this region, the conduction mechanism is governed by the space charge of the injected current, where the relationship between current and voltage typically follows a square or higher power law.

We fitted the forward I–V curve of the polycrystalline device according to the Poole−Frenkel model, as shown in Figure 3b, which follows the equation [28]:

(2) $$J=AE\cdot exp\left(-\frac{B}{kT}\cdot E^{\frac{1}{2}}\right),\;ln\left(I/V\right)\propto V^{\frac{1}{2}}$$

where *J* is the current density, *E* is the electric field intensity, *A* and *B* denote constants, and *k* stands for the Boltzmann constant. The logarithm of the current-to-voltage ratio *ln*(*I/V*) exhibits a nearly linear relationship with *V*^1/2^, indicating the dominance of the Poole−Frenkel mechanism in the conduction process of polycrystalline devices. Based on this model, we hypothesize that defects within the bulk of polycrystalline devices capture electrons and, under the influence of an external field, reduce the energy barrier of the defect states, thereby facilitating the successful excitation of electrons into the conduction band.

Foglietti et al. [29] confirmed the ohmic contact at the CeO_2_/NSTO interface and indicated that the redox process occurs at the Pt/CeO_2_ interface. Therefore, we believe that the differences in the RS effect between single-crystal and polycrystalline devices are due to the differences in grain boundaries within the SDC film. To verify the above hypothesis, we will also utilize X-ray Photoelectron Spectroscopy (XPS) for validation.

### 3.5. XPS Analysis and Switching Mechanism Analysis

Using XPS to probe the surface stoichiometric differences of single-crystal and polycrystalline SDC20 thin films, the Ce 3*d* XPS spectra for both cases are depicted in Figure S3. The Ce 3*d* spectra were fitted using the spin-orbit splitting dipole for 3*d*_3/2_ and 3*d*_5/2_ orbitals [30]. During the fitting process, the intensity ratio of the 3*d*_3/2_ to 3*d*_5/2_ peaks was fixed at 1.5 (with the 3*d*_3/2_ core energy level defined as u and the 3*d*_5/2_ core energy level defined as v), and a spin-orbit splitting binding energy difference Δ = 18.514 eV was employed. Additionally, five pairs of spin-orbit split peaks were fitted. Among these pairs, v_0_ and u_0_, v′ and u′, are associated with Ce^3+^, while v and u, v″ and u″, and v′′′ and u′′′ are associated with Ce^4+^. The fitting data are provided in Table S2. By calculating the ratio of the Ce^3+^ to Ce total peak areas, the Ce^3+^/Ce_tol_ values for the single-crystal and polycrystalline SDC20 films were determined to be 14% and 12.3%, respectively, as shown in Figure S3a. The fitting results indicate that, at near-surface positions, single-crystalline SDC20 thin films possess a greater abundance of oxygen vacancies compared to polycrystalline SDC20 thin films.

**Figure 4:**
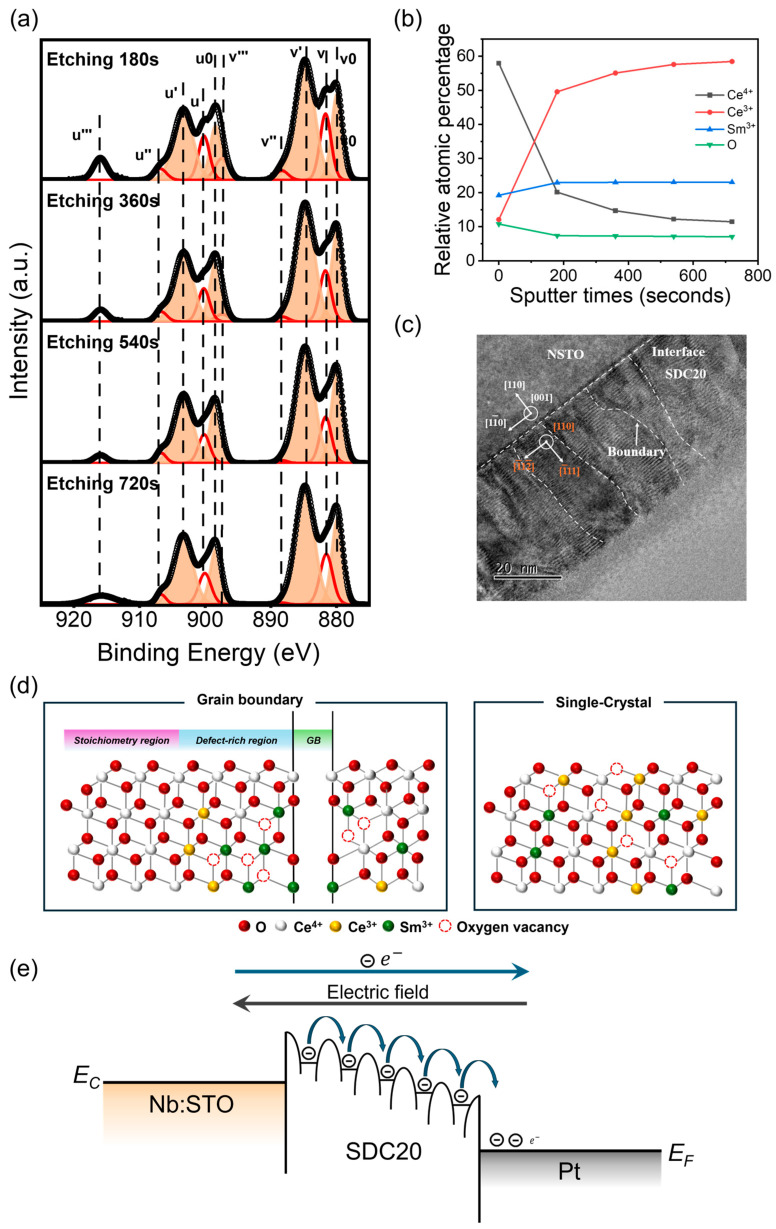
(**a**) The Ce 3*d* XPS spectra of polycrystalline SDC thin films at various etching depths. (**b**) Elemental analysis of polycrystalline SDC thin films at different etching depths. (**c**) TEM images of polycrystalline SDC thin films. (**d**) Schematic illustration of oxygen vacancy distribution within the bulk of SDC20 thin film. (**e**) Conduction mechanism analysis of the polycrystalline device.

However, in most studies, polycrystalline films typically exhibit a higher concentration of oxygen vacancies compared to single-crystalline films due to the presence of grain boundaries. Therefore, we conducted XPS analysis at various etching depths for both single-crystalline and polycrystalline films and plotted the variation of each element’s proportion with their etching depth. As shown in Figure 4a, after etching, a higher concentration of Ce^3+^ is exposed on the surface of the polycrystalline film. By calculating the peak areas, the proportions of each element can be determined. As depicted in Figure 4b, the proportion of Ce^3+^ increases with the etching depth. This increase is attributed to the unavoidable reduction reactions during the etching process and the exposure of Ce^3+^ from the bulk. Similarly, we performed the same characterization on the single-crystalline film. As shown in Figure S3b,c, etching also exposes more Ce^3+^ in the single-crystalline film. However, the proportion of Ce^3+^ stabilizes with continued etching, confirming that the increase in Ce^3+^ in the polycrystalline film during etching is due not only to reduction reactions but also to the exposure of Ce^3+^ from the bulk. At the same etching depth, polycrystalline SDC thin films exhibit a higher concentration of Ce^3+^ compared to single-crystal SDC thin films. This also confirms that polycrystalline SDC thin films have more oxygen vacancies in the bulk phase.

We propose the following explanation: The substitution of high-valence Ce^4+^ ions by low-valence Sm^3+^ ions lead to the generation of many defects, namely oxygen vacancies, to maintain charge neutrality. Under the conditions of 20 mol% doping, the coordination number of Ce is greater than that of Sm, resulting in a greater tendency for oxygen vacancies to be surrounded by Sm^3+^ [31]. Research conducted by Lei et al. has reported that trivalent dopant ions tend to aggregate at grain boundaries [32], thereby leading to a higher concentration of oxygen vacancies near these boundaries, while relatively fewer oxygen vacancies are observed at near-surface positions. Dou et al.’s work demonstrated that conductive channels form near grain boundaries [24]. This finding explains why polycrystalline devices exhibit lower resistance in the high resistance state (HRS) compared to single-crystalline devices (as shown in Figure 2e,h), corroborating the fact that oxygen vacancies accumulate near grain boundaries.

In summary, the speculation that the conduction mechanism of polycrystalline SDC20 devices conforms to the Poole−Frenkel model appears to be valid. TEM results (Figure 4c) indicate the presence of numerous dislocations within the film, where grain boundaries containing abundant dislocation cores form a common [110] tilt axis, consistent with reported findings [25]. In combination with the TEM results, the accumulation of many oxygen vacancies at grain boundaries (as shown in Figure 4d) captures electrons during the conduction process. Under the influence of an external field, electrons are released from these defects, thereby successfully transitioning to the conduction band (as shown in Figure 4e).

## 4. Conclusions

In summary, we successfully fabricated single-crystalline and polycrystalline SDC films using PLD technology. These films were employed as RS layers to construct Pt/SDC20/NSTO devices, and their RS performance was studied. The single-crystalline device exhibited the best resistive switching performance, maintaining stable resistance states over 500 switching cycles with a switching ratio of 10^2^. In contrast, the polycrystalline device demonstrated poorer RS properties, attributed to the accumulation of Sm at grain boundaries, which generated numerous defects that captured electrons, making electron conduction reliant on high-electric fields. This viewpoint was confirmed by XPS and I–V curve fitting. This work highlights the negative impact of grain boundaries in doped systems and provides new insights for the development of advanced memristor devices.

## Data Availability

The raw data supporting the conclusions of this article will be made available by the authors on request.

## Supplementary Materials

The following supporting information can be downloaded at: https://www.mdpi.com/article/10.3390/ma17133360/s1, Figure S1: (a) XRD patterns of SDC20 powder. (b) The shift of the SDC (220) orientation peak.; Figure S2: SEM images of the single-crystal (a) and polycrystalline (b) SDC20 film. Figure S3: (a) The Ce 3*d* XPS spectra of single-crystal and polycrystalline SDC thin films. (b) Elemental analysis of single-crystal SDC thin films at different etching depths. (c) The Ce 3*d* XPS spectra of polycrystalline SDC thin films at various etching depths. Table S1: Table of ICP results for SDC.; Table S2: Comparison of fitting results of each component of Ce 3*d* XPS spectra of SDC thin films.
