# Supplementary material for: Investigation of Grain Boundary Effects in Sm0.2Ce0.8O2−x Thin Film Memristors
Source: Materials (Basel). 2024 Jul 8;17(13):3360. doi: 10.3390/ma17133360 (PMC11243247; doi:10.3390/ma17133360)
Supplement: Supplementary file 1 [file materials-17-03360-s001.zip › materials-3067679-supplementary.pdf]

Supporting Information

Element	wt%	stoichiometric ratio
Sm	17.19	0.198
Ce	65.49	0.802

Table S1. Table of ICP results for SDC20.

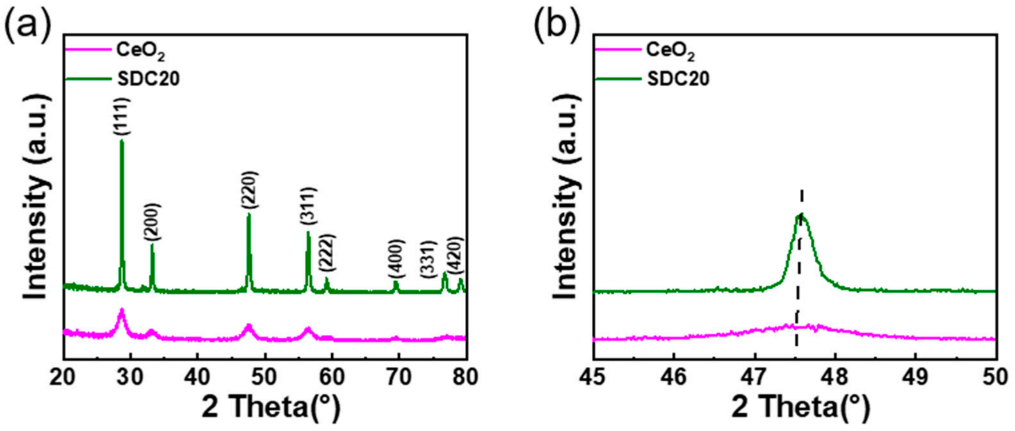

Figure S1. (a) XRD patterns of SDC20 powder. (b) The shift of the SDC (220) orientation peak.

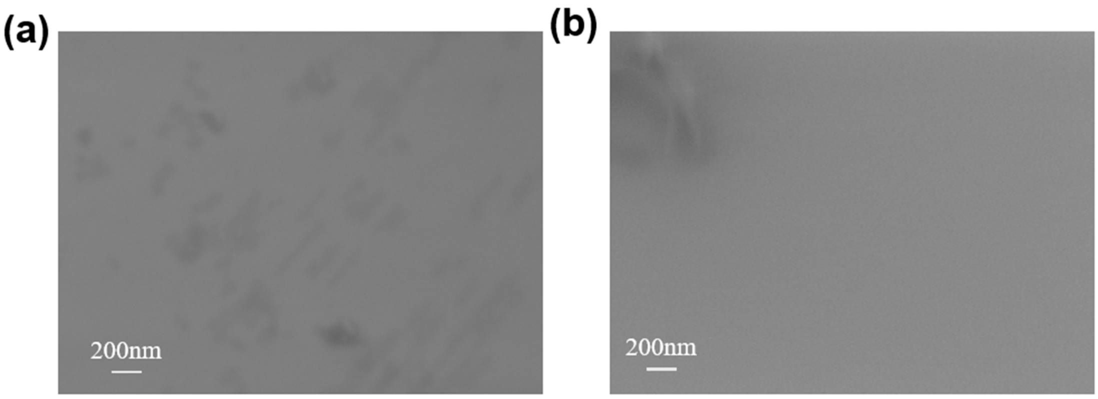

Figure S2. SEM images of the single-crystal (a) and polycrystalline (b) SDC20 film.

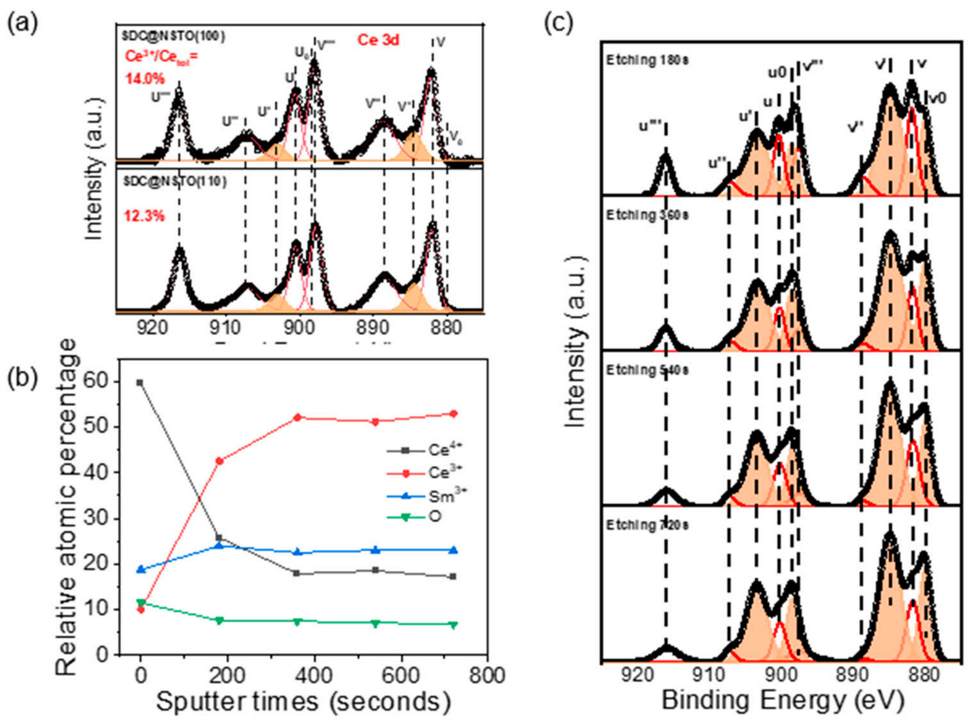

Figure S3. (a) The Ce 3d XPS spectra of single-crystal and polycrystalline SDC thin films. (b) Elemental analysis of single-crystal SDC thin films at different etching depths. (c) The Ce 3d XPS spectra of polycrystalline SDC thin films at various etching depths.

Single-crystal SDC			Fitting results		
Ce $3d_{5/2}$	v'''	v''	v'	v	v_0
Binding energy (eV)	897.95	888.7	884.44	882.19	879.62
FWHM	1.89	4	2.88	1.96	1.14
Ce $3d_{3/2}$	u'''	u''	u'	u	u_0
Binding energy (eV)	916.47	907.21	903.01	900.61	898.13
FWHM	2.29	4	2.88	1.97	1.14
**Polycrystalline SDC**			**Fitting results**		
Ce $3d_{5/2}$	v'''	v''	v'	v	v_0
Binding energy (eV)	897.81	888.59	884.43	882.03	879.94
FWHM	1.92	4	2.49	1.96	2

Ce $3d_{3/2}$	u'''	u''	u'	u	u_0
Binding energy (eV)	916.32	907.11	903.03	900.55	898.85
FWHM	2.37	4	2.48	1.96	2

Table S2. Comparison of fitting results of each component of Ce 3d XPS spectra of SDC thin films.
